# Prof. Miner’s Condition

**Published:** 1880-11

**Authors:** 


### PROF. MINER'S CONDITION.

WE are gratified to be able to announce that, since our last issue, Dr. Miner's condition has improved, and at times encouragement is felt in regard to his ultimate recovery. He is able to sit up a portion of the day. His convalescence will be slow, and many months will elapse before his strength will be sufficiently restored to enable him to give the profession the benefit of his large experience and acknowledged ability. Our best wishes go forth for our late editorial confrere, whose weary hours of confinement may be cheered with the assurance that the profession join in expressions of deep and sincere regard, as well as of anxious solicitude.

